# Haplotype Detection from Next-Generation Sequencing in High-Ploidy-Level Species: 45S rDNA Gene Copies in the Hexaploid *Spartina maritima*

**DOI:** 10.1534/g3.115.023242

**Published:** 2015-11-03

**Authors:** Julien Boutte, Benoît Aliaga, Oscar Lima, Julie Ferreira de Carvalho, Abdelkader Ainouche, Jiri Macas, Mathieu Rousseau-Gueutin, Olivier Coriton, Malika Ainouche, Armel Salmon

**Affiliations:** * UMR CNRS 6553 Ecobio, OSUR (Observatoire des Sciences de l’Univers de Rennes), University of Rennes 1, Bât 14A Campus Scientifique de Beaulieu, 35 042 Rennes Cedex, France; † Biology Centre ASCR, Institute of Plant Molecular Biology, Branišovská 31, České Budějovice, CZ-37005, Czech Republic; ‡ UMR Institut de Génétique, Environnement et Protection des Plantes, Institut National de la Recherche Agronomique, BP35327, 35653 Le Rheu Cedex, France

**Keywords:** poaceae, duplication, paralogy, polyploidy, bioinformatics

## Abstract

Gene and whole-genome duplications are widespread in plant nuclear genomes, resulting in sequence heterogeneity. Identification of duplicated genes may be particularly challenging in highly redundant genomes, especially when there are no diploid parents as a reference. Here, we developed a pipeline to detect the different copies in the ribosomal RNA gene family in the hexaploid grass *Spartina maritima* from next-generation sequencing (Roche-454) reads. The heterogeneity of the different domains of the highly repeated 45S unit was explored by identifying single nucleotide polymorphisms (SNPs) and assembling reads based on shared polymorphisms. SNPs were validated using comparisons with Illumina sequence data sets and by cloning and Sanger (re)sequencing. Using this approach, 29 validated polymorphisms and 11 validated haplotypes were reported (out of 34 and 20, respectively, that were initially predicted by our program). The rDNA domains of *S. maritima* have similar lengths as those found in other Poaceae, apart from the 5′-ETS, which is approximately two-times longer in *S. maritima*. Sequence homogeneity was encountered in coding regions and both internal transcribed spacers (ITS), whereas high intragenomic variability was detected in the intergenic spacer (IGS) and the external transcribed spacer (ETS). Molecular cytogenetic analysis by fluorescent *in situ* hybridization (FISH) revealed the presence of one pair of 45S rDNA signals on the chromosomes of *S. maritima* instead of three expected pairs for a hexaploid genome, indicating loss of duplicated homeologous loci through the diploidization process. The procedure developed here may be used at any ploidy level and using different sequencing technologies.

Gene and genome duplications play an important role in the diversification of eukaryotic functions and the formation of new species (Ohno 1970; Wendel 2000). These common phenomena contribute to sequence heterogeneity at homologous loci in all plant (eukaryotic) genomes. Detection of the duplicated copies is essential to investigate species history and the evolutionary dynamics of genes. In this work, we focus on a multigene family widely used in evolutionary genetics, coding for ribosomal RNA (45S rDNA) in the context of a highly duplicated genome, the hexaploid *Spartina maritima* (Curtis) Fern. (Poaceae, Chloridoideae).

Polyploidy or whole genome duplication is a process that has played a major role in the evolution and the adaptation of many eukaryotes in both animals (Mable 2004; Van de Peer *et al.* 2009) and plants (Jiao *et al.* 2011; Levy and Feldman 2002; Soltis *et al.* 2009). Whole genome duplication is most often due to the formation of unreduced gametes during meiosis in the same species (autopolyploidy) or in an interspecific hybrid (allopolyploidy) (Ramsey and Schemske 1998). Autopolyploid species contain several homologous genomes, whereas allopolyploid species contain more or less divergent duplicated homeologous genomes.

The grass genus *Spartina* Schreb. belongs to the subfamily Chloridoideae (a poorly investigated group in genomics), where it forms a monophyletic lineage embedded in the paraphyletic *Sporobolus* genus, which recently led some authors to consider taxonomical inclusion of *Spartina* in genus *Sporobolus* (Peterson *et al.* 2014). The *Spartina* clade is particularly affected by recurrent polyploidization and/or interspecific hybridization events (Ainouche *et al.* 2008; Strong and Ayres 2013). It contains 13–15 species ranging from tetraploid (2n = 4x = 40) to dodecaploid (2n = 12x = 120, 122, 124) levels, with a basic chromosomes number x = 10. So far, no diploid *Spartina* species has been identified (Ainouche *et al.* 2012). The different *Spartina* species have evolved into two major lineages (Baumel *et al.* 2002): a tetraploid clade mainly consisting of American native species and a hexaploid clade, including New World and Old World species. Within this hexaploid clade, *Spartina maritima*, native to European Atlantic coasts and *Spartina alterniflora* Loisel., native to American east coasts, have naturally hybridized twice in the early 19^th^ century following introductions of the American species in Europe. Hybridization between these species led to the formation of two sterile F1 hybrids, *Spartina x townsendii* in the Bay of Southampton (England) (Groves and Groves 1880) and *Spartina x neyrautii* in the Basque region (France) (Foucaud 1897). Genome duplication of *S. x townsendii* gave rise to the highly fertile and invasive allopolyploid *Spartina anglica* C. E. Hubbard (2n = 12x = 120,122,124) (Marchant 1968). *S. anglica* colonized Western Europe as well as various regions (*e.g.*, Australia and China). This expansion has many ecological effects, with this species playing an important role in the salt marsh sediment dynamics. *S. anglica* is able to colonize mudflats and to accelerate sediment accretion, thus altering the characteristics of the colonized habitats (referred to as an “ecosystem engineer” species) (Ainouche *et al.* 2008 and references therein). Genome evolution analysis of *S. anglica* compared to its parents (*S. maritima* and *S. alterniflora*) offers a special opportunity to understand the genomic and adaptive mechanisms associated with the formation of a new species in the wild (Ainouche *et al.* 2012). With this perspective, knowledge of the parental genomes is essential.

Our study focused on the European parental species *S. maritima* (2n = 60), which has a genome size that is estimated to be 2C = 3.8 pg or ∼3700 MB (Fortune *et al.* 2008). This hexaploid species, probably of hybrid origin, is expected to contain three duplicated homeologous genomes (Fortune *et al.* 2007) that may have diverged in the past 10 mya (Rousseau-Gueutin *et al.* 2015). Sequence heterogeneity is then expected as a result of successive whole-genome duplications. This may be more complicated in gene families where both paralogs and homeologs may be encountered, such as the 45S ribosomal gene family, which is known for its highly dynamic evolution, most particularly in polyploids (see below). Because these genes are commonly used in phylogenetic studies, distinguishing paralogs and homeologs are critical.

The 45S rDNA unit is composed of a high number of transcription units (TU) per genome (>500 in plants) arranged in tandem repeats on one or several loci (Rogers and Bendich 1987; Prokopowich *et al.* 2003). Each unit contains three coding regions (18S, 5.8S, and 25S/26S) separated by two internal transcribed spacers (ITS-1 and ITS-2). The 18S and 25S coding regions are flanked by two external transcribed spacers or ETS (Figure 1) (Schaal and Learn 1988; Poczai and Hyvönen 2010). The 5′-ETS is subdivided into three regions: the ETS region I, which contains the TATA box corresponding to the transcription initial site (TIS); the ETS region II, which includes several and highly variable subrepeats; and the ETS region III, which is adjacent to the 18S coding region (Volkov *et al.* 1996). Each TU is separated from the other by an intergenic spacer (IGS) that contains the two ETS bordering the nontranscribed spacer region (NTS) composed of several repeats of 80 bp. The number of repeats and the length of each vary considerably among species (Poczai and Hyvönen 2010). The different repeats are known to undergo a process of homogenization by gene conversion leading to the observation of “concerted evolution” of these ribosomal genes (Nei and Rooney 2005). This homogenization limits the number and divergence of paralogous copies in this gene family, which has promoted its use in molecular phylogenies. The differential rates of variation along the transcriptional unit (the coding regions are more conserved than the spacers) allow phylogenetic inferences at different levels of the taxonomic hierarchy (Alvarez and Wendel 2003). In allopolyploid species, it was shown that the process of concerted evolution also affects homeologs, resulting in a preferential retention of one of the parental repeat copies, as demonstrated in polyploid cottons (Wendel *et al.* 1995) or tobacco (Kovarik *et al.* 2008). This process can occur rapidly after allopolyploid speciation, as evidenced in natural populations of allotetraploid *Tragopogon* formed in the past 80 years (Koh *et al.* 2010). In *Spartina*, the ITS region (including the 5.8S gene flanked by the ITS-1 and ITS-2) has been used to elucidate phylogenetic relationships among the different species (Baumel *et al.* 2002; Fortune *et al.* 2007), but studies on the evolutionary dynamics of the paralogous and homeologous copies are lacking in this system. The recently formed *Spartina* hybrid (*S. x townsendii*) and its allododecaploid derivative (*S. anglica*) exhibit parental additivity of the ITS regions inherited from their hexaploid parents (Baumel *et al.* 2001), and recent investigations in natural populations suggest that interlocus homogenization and/or homeolog loss may also occur in this region (D. Huska, I. J. Leitch, J. Ferreira de Carvalho, A. R. Leitch, A. Salmon, M. Ainouche, and A. Kovarik, unpublished data).

**Figure 1:**
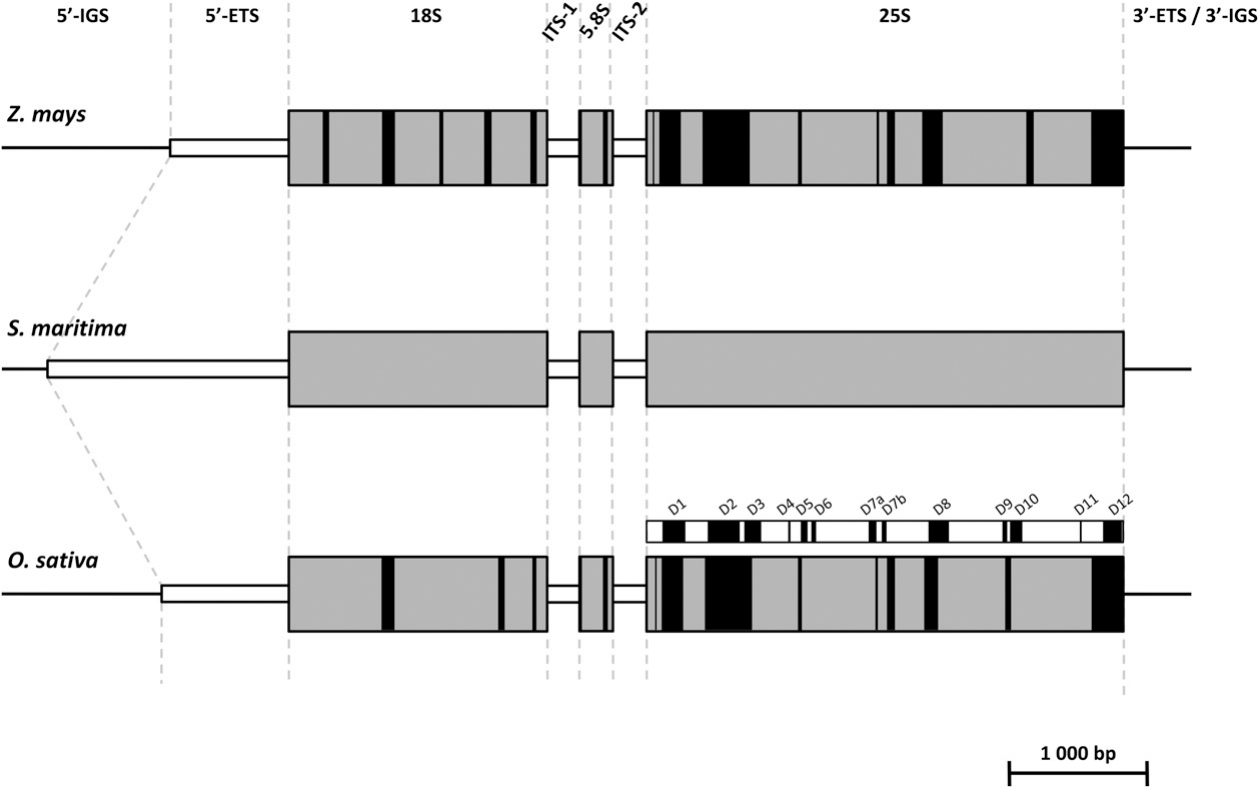
Schematic representation of the 45S ribosomal DNA in *Z. mays*, *S. maritima*, and *O. sativa*. The lines correspond to the intergenic spacers, the gray boxes correspond to the coding regions (18S, 5.8S, and 25S), and the white boxes correspond to the external and internal transcribed spacers (ETS, ITS-1, and ITS-2). Black boxes represent the regions presenting less than 90% identity between either *Z. mays* and *S. maritima* or *O. sativa* and *S. maritima*. The rice expansion segments (D1 to D12, represented in black) detected by Hancock and Dover (1988) and Kuzoff *et al.* (1998) are indicated above the rice 25S. The figure is drawn to scale.

Next-generation sequencing technologies (*e.g.*, pyrosequencing or sequencing by synthesis) offer powerful tools to generate suitable sequence read depth especially in nonmodel species where genomic resources were previously lacking (El-Metwally *et al.* 2014; Metzker 2009). Detection of different copies expected in these genomes may be challenging, because individual gene duplications (resulting in paralogs in both diploid and polyploid genomes) usually add an additional layer of complexity (Ainouche *et al.* 2012). Detection and analysis of homeologs is central to polyploidy research, and several studies have focused on the detection of different copies in polyploid genomes such as in *Glycine* (Ilut *et al.* 2012), *Gossypium* (Flagel *et al.* 2008; Salmon *et al.* 2010), *Coffea* (Combes *et al.* 2011), or *Triticum* (Akhunova *et al.* 2010) species. In these systems, the diploid parental (or related) representatives are known and can be used to identify duplicated homeologs in the allotetraploids. The strategy developed in these studies is to assemble NGS datasets using parameters adapted to optimize recovery of paralogous and homeologous copies. Contigs obtained are then compared with diploid parental genomes using species-specific polymorphic sites.

The detection of different copies in polyploid species where the parents are not identified or extinct still requires the development of adapted tools. The goal of this study is to develop a bioinformatic pipeline to detect the different copies within a set of NGS reads from a highly polyploid species without any reference parental diploid genome. We developed this pipeline to explore the heterogeneity of the coding and noncoding regions of the highly repeated 45S unit of the hexaploid *Spartina maritima*.

The strategy was to: (i) identify single nucleotide polymorphisms (SNPs) and indels among reads from the hexaploid genome and (ii) assemble reads based on shared polymorphisms (after removing putative sequencing errors) to distinguish the different copies from reads. Polymorphisms were validated using comparisons with Illumina sequence data sets and by cloning and Sanger (re)-sequencing. The method presented here can be used to identify duplicated copies from any sequenced regions of polyploid or diploid genomes.

## Materials and Methods

### Plant material sequencing

Samples from *Spartina maritima* were collected at the Etel river estuary (Morbihan, France). Plants were transplanted and maintained in controlled conditions in the greenhouse (University of Rennes 1, France). Total genomic DNA was isolated from fresh leaf tissue with the Nucleospin Plant II (Macherey-Nagel) extraction kit following the manufacturer’s instructions. One run of *S. maritima* DNA was sequenced at the Functional and Environmental Genomics platform (Biogenouest, OSUR Rennes, France) using a 454 GS FLX Titanium pyrosequencer (Life Sciences, Roche) that generated 999,229 reads with an average length of 377.0 bp. Illumina genomic reads (172,528,550 reads of 100 bp length; 500-bp paired ends) from *S. maritima* (from the same population) were also used for mapping and validation or correction of the detected SNPs. Genome coverage and 45S rDNA copy number estimates are presented in Supporting Information, Table S1 for both Roche-454 and Illumina Whole Genome Shotgun sequencing.

### Assembly and annotation of the 45S rDNA region

**Figure 2:**
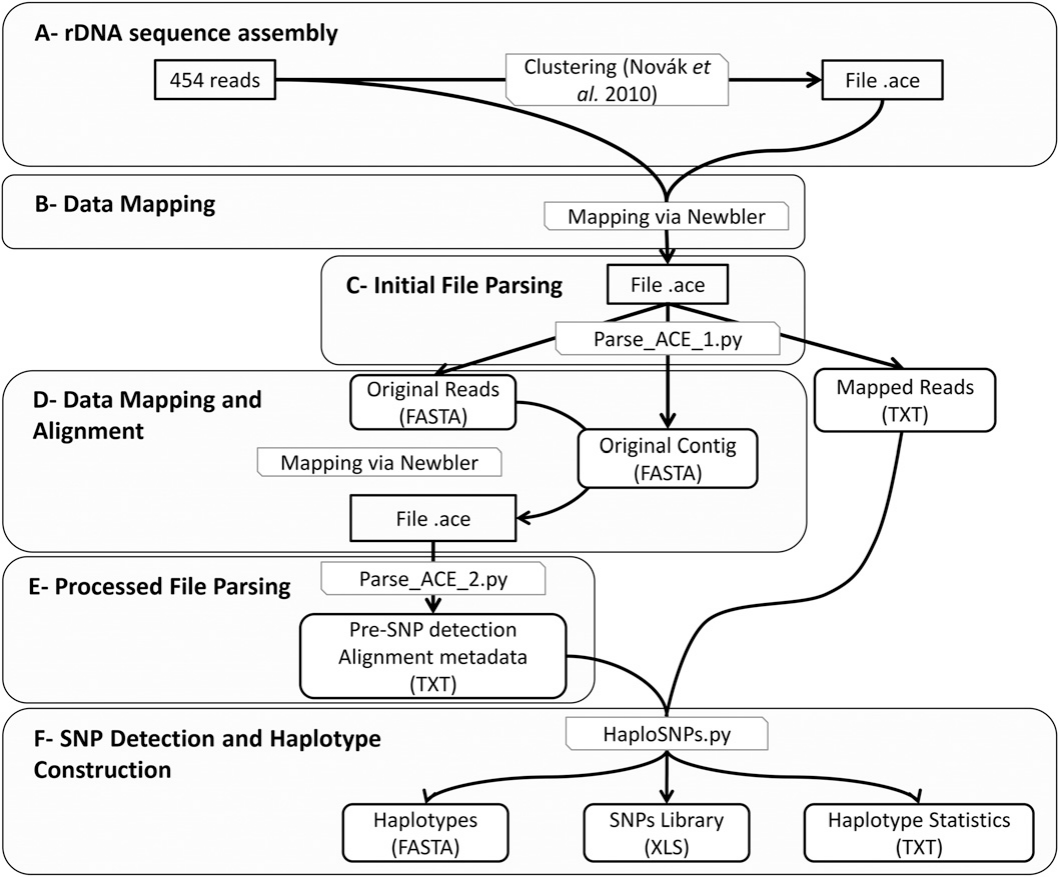
Overall workflow for haplotype construction using Roche-454 data.

We first analyzed Roche-454 genomic reads of *S. maritima* (Figure 2A) using the computational pipeline developed by Novák *et al.* (2010). This pipeline uses graph-based clustering of sequence reads sharing mutual sequence similarities (minimum percent identity: 90%; minimum overlap: 55% of the shorter sequence length) to detect groups of frequently overlapping reads representing genomic repeats. In addition, it performs contig assembly within identified clusters and provides information aiding in repeat annotation. The cluster representing *S. maritima* 45S rDNA was identified by similarity searches against various available 45S rDNAs of Angiosperms, and an 8456 bp-long contig was retrieved from the assembled reads. Roche-454 genomic reads were then mapped on this contig to increase the number of reads. This first mapping procedure (Figure 2B) was performed using the GSMapper tools from Newbler (ml = 100 bp; mi = 95%). Annotation of the different 45S domains was performed by aligning the *S. maritima* contig with *Oryza sativa* and *Zea mays* 45S genes available on the NCBI database (GenBank IDs: X00755.1, AF169230, M11585.1 and X03989, NR_036655.1, AF019817, NR_028022.2, respectively) using BLASTn with default parameters for the alignment algorithm Megablast (Table 1) (Altschul *et al.* 1997). The *Oryza sativa* genome was downloaded from www.phytozome.net (v. 204) to locate the 5′-ETS region. To identify this region, we followed the approach of Barker *et al.* (1988), who identified the repeat unit of the rDNA intergenic region and the transcription initial site.

**Table 1:** Identification of the different rDNA regions in *S. maritima* after comparisons with *Oryza sativa* and *Zea mays*

Species		5′-ETS	18S	ITS-1	5.8S	ITS-2	25S
*S. maritima*	Length (in bp)	1754	1812	226	167	206	3391
	start-end region	392–2145	2146–3957	3958–4183	4184–4350	4351–4556	4557–7947
	GC%	50.9	50	53.5	52.7	49.5	55.4
*O. sativa*	Length (in bp)	966	1812	198	167	215	3377
	GC%	73.0	51.3	72.7	58.1	79.1	59.4
	GenBank IDs	—*^a^*	X00755.1	AF169230	AF169230	AF169230	M11585.1
	Blastn results (Identity, e-value)	—	97%, 0.0	—	94%, 2e-69	—	93%, 0.0
*Z. mays*	Length (in bp)	834	1809	213	164	220	3385
	GC%	70.0	51.0	70.4	56.7	73.2	58.7
	GenBank IDs	X03989	NR_036655.1	AF019817	AF019817	AF019817	NR_028022.2
	Blastn results (Identity, e-value)	—	96%, 0.0	—	94%, 2e-68	—	93%, 0.0

The length, position, and GC content of each domain are presented. Genbank accession numbers of the *O. sativa* and *Z. mays* sequences used, as well as the percentage of identity and e-value between these and the *S. maritima* sequences, are mentioned.

a 5′-ETS was detected using *O. sativa* genome (v. 204).

### SNPs detection and sequencing error removal

To detect SNPs, we developed a pipeline allowing the parsing of “.ACE” alignment files (Figure 2C) using the Ace.py program from biopython (http://biopython.org/) and custom python script for editing homopolymer-driven false-positive SNPs. This step produced three files containing original reads, the original contig, and the list of the original reads (Figure 2D). This pipeline uses a mapping step with GSMapper (v.2.6, Roche; default parameters: ml = 40 bp; mi = 90%) to enhance alignments and ease SNP detection. The newly generated “.ACE” file is parsed to extract the aligned reads (Figure 2E). To remove false-positive SNPs caused by sequencing errors (within reads, and at the first or last position), the first five and the last five nucleotides of each read, as well as nucleotides with frequency lower than 20% were not considered. SNPs present at least in one-fifth of aligned reads were then considered true positives. SNPs present in homopolymeric region (more than four identical nucleotides) were not considered. The nature of the detected polymorphisms and their frequency are stored as output results (Figure 2F). Visual checking of the detected and not considered false positives was done using the Tablet software (Milne *et al.* 2009) for “.ACE” alignment files and with the Jalview software (Waterhouse *et al.* 2009) for “.FASTA” files.

### Haplotype detection

**Figure 3:**
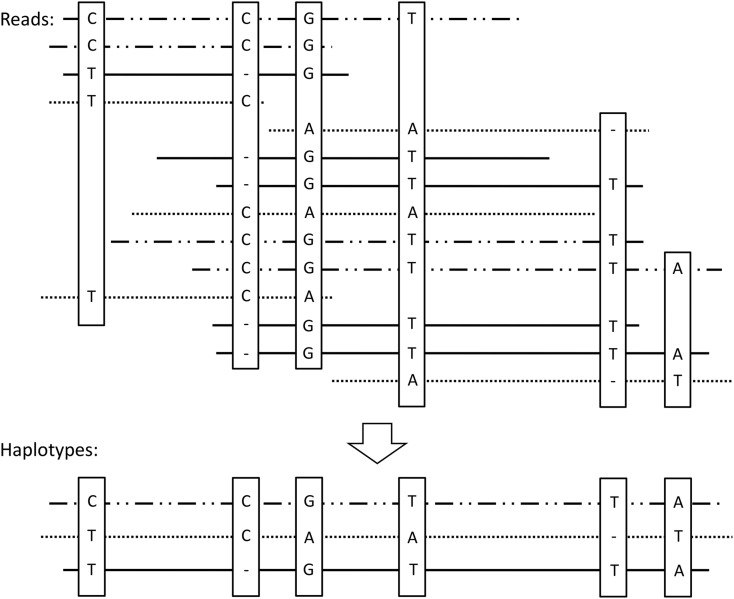
Representation of a read assembly. After SNP detection and false-positive correction, all reads displaying the same SNP (represented with the same line) are assembled to create a haplotype that corresponds to a consensus sequence of maximal size.

Haplotypes were detected after the SNP detection by identifying and assembling reads sharing the same SNPs. This assembly was performed by comparing each read to all the others and by screening all the SNPs present in overlapping regions. If all the SNPs are identical in this region, the considered reads are assembled to create a haplotype with a maximum size. A unique haplotype is then a sequence characterized by multiple polymorphic sites corresponding to the same and unique allele (Figure 3). However, for each alignment, it was necessary to define different windows corresponding to local alignments for counting haplotypes. The windows exhibiting at least one SNP were selected to count the number of locally aligned haplotypes. Two output files allow the dataset visualization: (1) a “.FASTA” file that contains the consensus sequence with the different aligned haplotypes and (2) a tabulated file describing SNPs positions and their nature. A third output file is created during these processes and contains information about the assembly, the name of the contig, the initial and final lengths of the contig obtained after the correction of sequencing errors, the number of reads used to assemble the contig, and the different haplotypes. This file contains the number of SNPs and their position, the number of haplotypes, and their coverage (mean, SD, and C.I. of 95%). The last information contained in this file is the number of haplotypes for each window.

The SNP and haplotype detection programs described above were incorporated into a single pipeline that includes four adjustable parameters: (i) the read trimming length corresponding to the number of nucleotides at the beginning and the end of each read not to consider in the SNP detection step; (ii) the minimum read depth corresponding to the number of reads required to detect SNP from; (iii) the SNP detection threshold corresponding to the minimal proportion of reads displaying a putative polymorphism; and (iv) the minimal number of shared SNPs to assemble two reads into a haplotype. The minimum depth of the analyzed rDNA contig was 61. Consequently, the minimum read depth parameter was fixed to 60. The other default parameters used in our pipeline were as follows: read trimming length = 5; SNP detection threshold = 20; number of shared SNPs = 1.

### Cloning and sequencing of polymorphic 45S rDNA regions

For validating the SNP and haplotype detection pipeline we developed, a subset of the detected SNPs and haplotypes was targeted for molecular cloning and (re)-sequencing (Sanger method). We have designed four pairs of primers using Primer 3, v. 1.1.4 (Rozen and Skaletsky 2000) (Table 2) flanking variable regions detected with pyrosequencing (5′- IGS/ETS, ITS-1, 25S and 3′- ETS/IGS). All amplifications were carried out in a 50 μL reaction mixture containing 45 µl of Platinum PCR SuperMix High Fidelity (Invitrogen), 2 μl of each 5 μM primer, and 1 μl of DNA (50 ng). The PCR amplification conditions were as follows: 2 min of DNA denaturation at 94° followed by 30 cycles of 30 sec at 94°, 20 sec at 58°, and 30 sec at 68° for each cycle, followed by 8 min final extension at 68°. PCR products were purified with the NucleoSpin Gel and PCR Clean-up (Macherey-Nagel). Cloning reaction was performed with TOPO TA Cloning Kits for sequencing (Invitrogen) using 2 μl of PCR purified products, 1 µl of salt solution (1.2 M of NaCl, 0.06 M of MgCl_2_), 2 µl of water, and 1 µl of plasmid vector pCR4Blunt-TOPO (Invitrogen). The reactions were incubated for 5 min at room temperature. Transformations were realized using electro-competent *Escherichia coli* DH5α and Gene Pulser Xcell Electroporation System (BioRad); 18 µl of water was added to 6 µl of TOPO cloning reaction. Transformations were done in 0.2 cm cuvettes with 2 µl of DNA added to 40 µl of ElectroMax DH5α Competent Cells (Invitrogen) incubated 1 min on ice and pulsed at 2.5 kV, 25 µF, and 200 Ω. After the addition of SOC medium (1 ml), the cell suspension were incubated at 37° for 1 hr; 25–100 µL of each transformation culture was plated onto LB Agar with ampicillin (100 mg/ml) and incubated overnight in liquid medium at 37° (LB Broth and 100 mg/ml of ampicillin). Plasmids were purified with the Pure Yield Plasmid Miniprep System (Promega) and sequenced using T7 and T3 primers to sequence the samples (16–24 clones for each region) with 3730XL DNA sequencer technology (Sanger method) from both ends. For each region, the different sequences were aligned with MAFFT (v 6.864b) (Katoh and Toh 2010). All sequences were cleaned (plasmid deletion) and the sequencing errors were visually corrected using chromatographs.

**Table 2:** Primers designed to amplify and sequence the 45S ribosomal DNA in *S*. *maritima*

Primer	Sequence	Primer pair	Amplicon Size	Region
ASribo_1_FP1	ACACGACTGGGTTTAGTCCG	FP1/RP1	579	5′- IGS/ETS
ASribo_1_RP1	AGGCCAGGTTTAGTCCGTTT			
ASribo_2_FP1	AAACGGACTAAACCTGGCCT	FP1/RP2	720	5′-ETS
ASribo_2_RP2	CTATTTTCAGAGGGGGAGGG			
ASribo_5_FP1	TGTCGTGACCCAAACAAAAA	FP1/RP2	725	ITS-1/5.8S/ITS-2
ASribo_5_RP2	CGATTCTCAAGCTGGGCTAC			
ASribo_6_FP1	AGACATTGTCAGGTGGGGAG	FP1/RP2	752	25S
ASribo_6_RP2	AAAGGCCACTCTGCCACTTA			

### SNP validation and haplotype estimation with Illumina data

To compare pyrosequencing and cloning SNPs to Illumina SNPs, we mapped 1,263,153 Illumina paired reads with an average read depth of 7886 (SE = 273.84) on the rDNA sequence consensus of *S. maritima* using Bowtie2 (score-min: G, 52, 8) (Langmead and Salzberg 2012). These “score-min” parameters were included in the minimum score function f(x)= 52 + 8 * ln(x), where *x* corresponds to the read length. This function corresponds to a minimum of 87.06–90.30% of identity between the mapped reads and the reference sequence for reads with a length of 80–120 bp. The “.SAM” file created by Bowtie2 was converted in a “.PILEUP” format using the Samtools software suite (Li *et al.* 2009). We have detected the different SNPs within the Illumina data using custom python scripts (minimum read depth = 30; SNP detection threshold = 2, corresponding to nucleotides that are present more than two out of 100 times per position). To estimate the relative presence of each haplotype, Illumina reads were mapped on previously detected haplotypes (∼100 bp for each region including 5′-ETS/IGS, 18S, ITS-1 and 25S) using Bowtie2 (score-min: L, 100, 0; f(x)= 100 + 0 * x where x = length of read). Results obtained were filtered using custom python scripts to identify the number of reads mapped with 100% of identity.

### Chromosome preparation and fluorescence *in situ* hybridization

*In situ* hybridization was performed on mitotic chromosomes from *S. maritima* roots. Root tips with a length of 0.5–1.5 cm were treated in the dark with 0.04% 8-hydroxiquinoline for 2 hr at 4°, followed by 2 hr at room temperature to accumulate metaphases, then fixed in ethanol–acetic acid (3:1, *v/v*) for 48 hr at 4°, and stored in ethanol 70% at −20° until required. After washing in 0.01 M enzyme buffer (citric acid–sodium citrate, pH 4.5) for 15 min, the prepared roots were digested in a solution of 5% Onozuka R-10 cellulase (Sigma) and 1% Y23 pectolyase (Sigma) at 37° for 30 min. The root tips were then washed with distilled water for 30 min. A root tip was transferred to a slide and macerated with a drop of 3:1 fixation solution. After air-drying, slides with good metaphase chromosome spreads were stored at −20°. Fluorescence *in situ* hybridization was carried out using the ribosomal probe pTa 71 (Gerlach and Bedbrook 1979), which contained a 9-kb *Eco*RI fragment of rDNA repeat unit (18S-5.8S-25S genes and spacers) isolated from *Triticum aestivum*. The pTa 71 probe was labeled by random priming with biotin-14-dUTP (Invitrogen, Life Technologies). Chromosome preparations were incubated in RNase A (100 ng/µl) and pepsin (100 mg/ml) in 0.01 M HCl, and fixed with paraformaldehyde (4%). Chromosomes were denatured in a solution of 70% formamide in 2X SSC at 70° for 2 min, dehydrated in an ethanol series (70%, 90%, and 100%), and air-dried. The hybridization mixture, consisting of 50% deionized formamide, 10% dextran sulfate, 2X SSC, 1% SDS, and labeled probe (200 ng per slide), was denatured at 92° for 6 min and transferred to ice. The denatured probe was placed on the slide and *in situ* hybridization was carried out overnight in a moist chamber at 37°. After hybridization, slides were washed for 5 min in 50% formamide in 2X SSC at 42°, followed by several washes in 4X SSC-Tween. The biotinylated probe was immunodetected by Texas Red avidin DCS (Vector Laboratories). The chromosomes were mounted and conterstained in Vectashield (Vector Laboratories) containing 2.5 µg/ml 4’,6-diamidino-2-phenylindole (DAPI). Fluorescence images were captured using a CoolSnap HQ camera (Photometrics, Tucson, AZ) on an Axioplan 2 microscope (Zeiss, Oberkochen, Germany) and analyzed using MetaVue (Universal Imaging Corporation, Downingtown, PA).

### Data availability

The S. *maritima* rDNA consensus sequence and haplotype sequences are available at NCBI under the KT874468 to KT874488 accession numbers.

## Results

### Analysis and annotation of the 45S rDNA region

The contig obtained with the clustering approach was 8456 nucleotides long; it was assembled from 3219 reads with an average length of 377.0 bp. Genomic DNA reads of *S. maritima* were then mapped to the contig with GSMapper (with the following parameters: ml = 100 bp; mi = 95%) (Margulies *et al.* 2005), resulting in a total of 4014 aligned reads with an average length of 506.8 nucleotides. This step allowed us to build a 45S rDNA reference sequence totaling 8464 bp. The different regions of the 45S rDNA of *S. maritima* were annotated by homology searches using rDNA sequences from Poaceae species (*Zea mays*, *Oryza sativa*) available in GenBank (NCBI) and by motif searches for the 5′-ETS region (Table 1). The beginning of the 5′-ETS region was identified using the TATA-box (5′-TATATTAGGGGG-3′) motif at position 395, corresponding to the expected plant TATA-box motif (5′-TATA(G)TA(N)GGGGG-3′), as found in several species such as *Zea mays*, *Oryza sativa*, or *Arabidopsis thaliana* (Fan *et al.* 1995; Zentgraf *et al.* 1998). To confirm this position, a dot plot of the 5′-IGS/ETS region against itself was performed. Regions 2 and 3 of the 5′-ETS (Volkov *et al.* 1996, 1999) were also detected. We found that regions 2 and 3 of the 5′-ETS totaled 1600 bp. This confirms the total length of the 5′-ETS of *S. maritima* (1754 bp), which is approximately two-times larger than the 5′-ETS of *Z. mays* (825 bp; X03989) and *O. sativa* (966 bp). The *S. maritima* rDNA contig covers the whole coding region, spanning the 18S, 5.8S, and 25S, the ITS-1, ITS-2, 5′-ETS (Figure 1), and part of the IGS (5′-IGS: 391 bp; 52.9 GC% and 3′ETS/3′-IGS: 517 bp and 42.6 GC%) (Table 1). Interspecific comparisons of the three coding rDNA regions between maize and *Spartina* and between rice and *Spartina* indicate that nucleotide variation is distributed all along these regions (Figure 1).

### SNP validation in coding and noncoding regions

Using the mapped Roche-454 data, 34 SNPs or indels (insertion/deletion) were identified. These polymorphisms were localized both in coding (18S, 25S) and noncoding regions (5′-IGS, 5′-ETS, ITS-1 and 3′-ETS/IGS). Cloning and resequencing were performed and confirmed 10 of the 34 polymorphic sites (two substitutions, four variants including substitution and indel at the same position, and four indels) that had been detected using the mapping procedure. Twenty-nine SNPs and indels (including nine polymorphic sites found by cloning and (re)-sequencing) were subsequently validated using Illumina data (Table 3). Within the coding region, one indel and four SNPs were detected and validated using the Roche-454 and lllumina data in the 18S and 25S domains, respectively. In the noncoding regions, a substitution, the variant presenting substitution and indel at the same position, and two indels were validated using Illumina method in the 5′-IGS. In the 5′-IGS/ETS noncoding region, a total of 21 polymorphisms were detected using the mapped Roche-454 data, including nine substitutions and 12 indels (two of 4 bp) (Table 3). In this region, all the identified substitutions (except one G/T, position 1235) were validated using Illumina data. Within the ITS-1 region, the Roche-454 and Illumina data detected the same SNP (A/T/-) that was validated by (re)-sequencing. However, the indel was only found in the pyrosequencing data. In the 3′-ETS/IGS noncoding region, one indel using the Roche-454 data was not validated by Illumina data. Thus, the cloning-sequencing data as well as the Illumina reads substantiated 29 of the 34 polymorphisms previously identified in the Roche-454 reads. Four of the five invalidated variants were included in homopolymeric regions. The last indel was not found in the Illumina data, but cloning and sequencing data revealed variation in the chromatograph, suggesting either a sequencing error or a substitution.

**Table 3:** Nucleotide polymorphisms detected within coding and noncoding regions of *Spartina maritima* rDNA using the three methods (mapping method, cloning-sequencing Sanger method, and Illumina method)

Region	Mapping Method	Variants Validated	
		Cloning-Sequencing	Illumina
5′-IGS	6 (4)	3	4
5′-ETS	21 (21)	4	21
18S	1 (1)	—	1
ITS-1	1 (1)	1	1
25S	4 (2)	2	2
3′-IGS/ETS	1 (0)	—	0

The numbers between brackets correspond to the number of variants selected for haplotype reconstruction. Cloning and sequencing were not performed in the 18S and 3′-IGS/ETS regions.

### Haplotype detection

**Figure 4:**
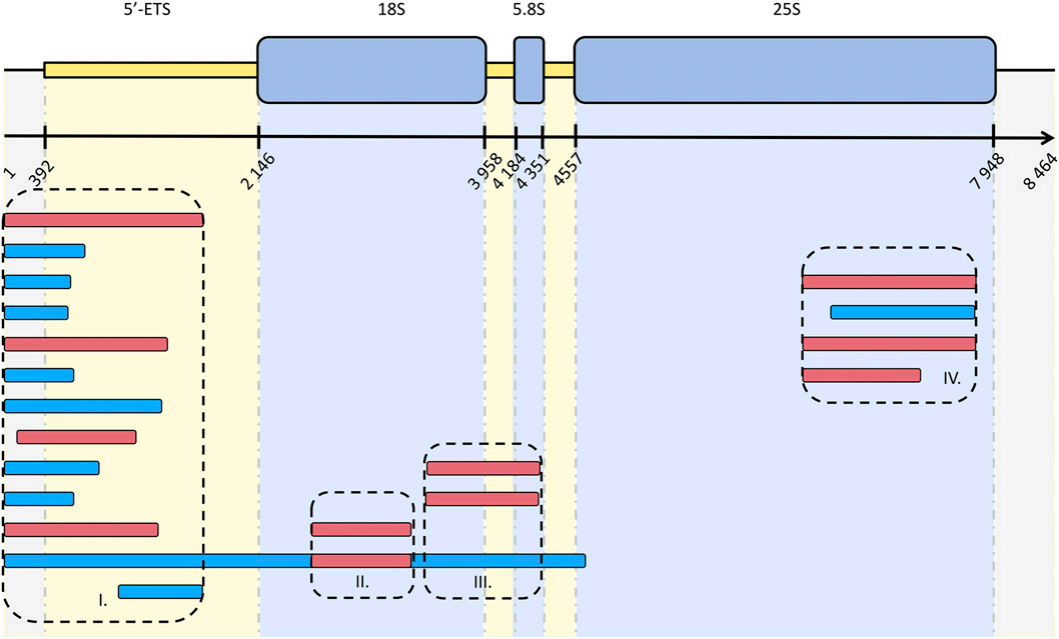
Schematic representation of haplotypes detected in *S. maritima* rDNA using our developed program (mapping data) and cloning data. The blue boxes represent the haplotypes detected only by the developed program. The red boxes represent the haplotypes detected by the developed program and validated by cloning and (re)-sequencing or Illumina data (*i.e.*, haplotypes in the block II). The different blocks (I to IV) correspond to regions of rDNA presenting at least two haplotypes.

After rDNA domain annotations and SNP detection and validation, the number of haplotypes for each region of the 45S unit was determined. In this study we retained only the Roche-454 haplotypes constructed with a minimum of 10× read depth. The five indels not validated by cloning and Illumina data were not considered. From the 29 polymorphic sites retained, 20 haplotypes with a mean length of 1197.70 bp (σ = 938.32; C.I._95%_ = 786.47–1608.93) were constructed using our program. These haplotypes were constructed from 69.35 reads on average (σ = 66.24; C.I._95%_ = 40.32–98.38). The 20 different haplotypes were localized on the different rDNA domains as shown in Figure 4. We did not identify any polymorphism in the 5.8S coding region, the internal transcribed spacer 2 (ITS-2), and the 3′ intergenic spacer (IGS). The 5′-IGS/ETS region was the most variable; we detected four variants in the 5′-IGS and 21 variants in the 5′-ETS. These SNPs allowed the construction of 13 haplotypes in this region (Figure 4, Block I). One indel was detected in the 18S coding region indicating the presence of two haplotypes (Figure 4, Block II). Within the ITS-1 we detected only one SNP indicating the presence of three haplotypes (Figure 4, Block III). Within the 25S, four haplotypes were constructed from two SNPs (Figure 4, Block IV). Finally, 20 haplotypes were obtained in total.

### Haplotype validation

To validate the developed program, the constructed Roche-454 haplotypes were compared with haplotypes obtained using the cloned sequences (16X to 24X both ends). Four regions were selected for these comparisons (5′-IGS/ETS, 5′-ETS, ITS-1/5.8S/ITS-2, and 25S). In the 5′-IGS/ETS noncoding region, seven polymorphisms were validated by cloning, including the indel block of 2 bp, one substitution, two deletions, and two substitutions/insertions/deletions. Using these polymorphisms, it was possible to validate three of nine Roche-454 constructed haplotypes. Cloning depth could explain the low number of haplotypes validated. In the 5′-ETS noncoding region, only one polymorphism was validated by the cloning approach, which confirms one haplotype (two haplotypes were detected with this variant using Roche-454 data) (Figure 4, Block I). In the ITS-1/5.8S/ITS-2, cloning-sequencing data validated the variant detected with the program, and two of three detected haplotypes were validated (Figure 4, Block III). In the 18S coding region, the two haplotypes detected with the program were validated using Illumina data (Figure 4, Block II). In the 25S coding region, the two SNPs detected using our program (developed on Roche-454 data) were found with cloning/(re)-sequencing data. Four haplotypes were found with the program and three were validated (Figure 4, Block IV).

### Haplotype proportion estimation

**Figure 5:**
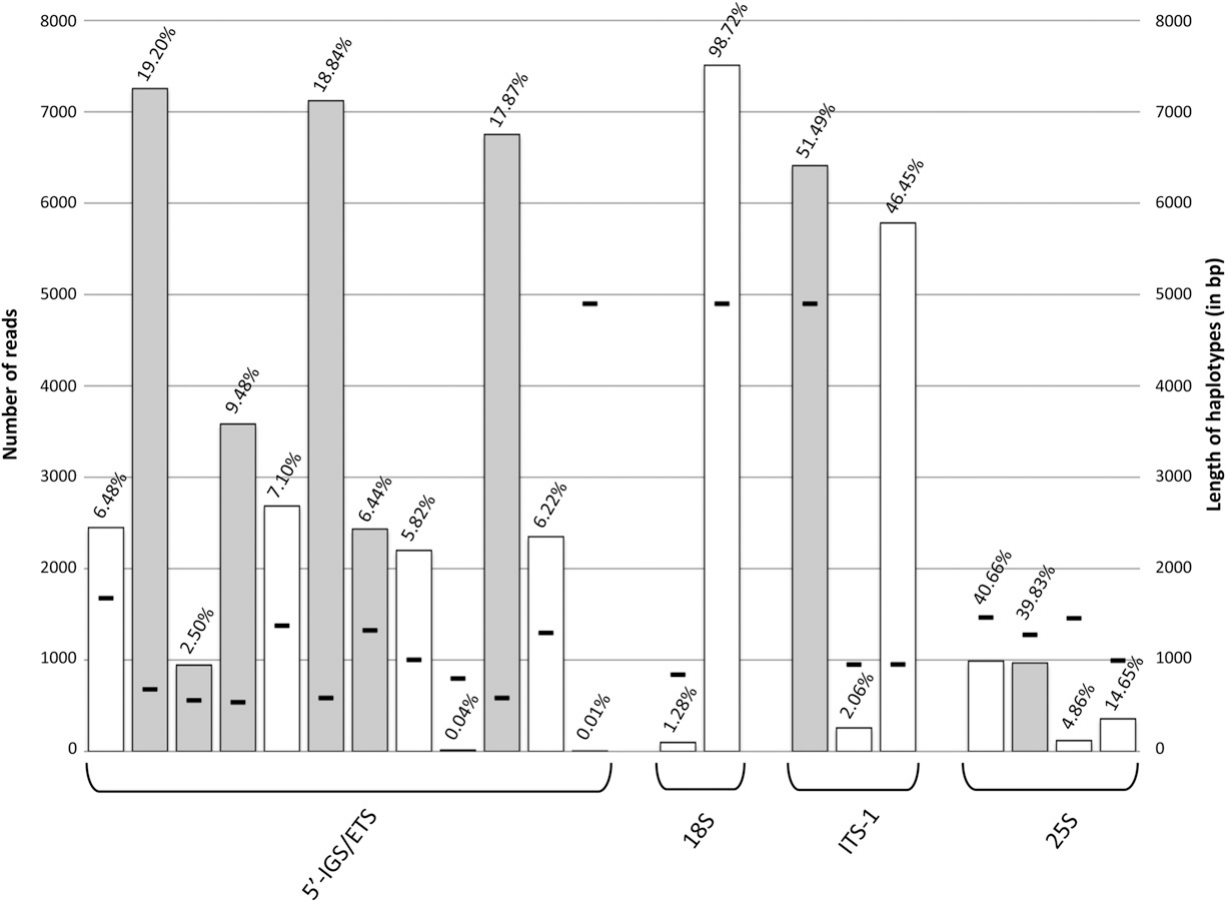
Number of reads mapped with 100% of identity on the different haplotypes for each region (5′-IGS/ETS, 18S, ITS-1, and 25S). The relative proportion of haplotype is presented above each histogram. White bars represent haplotypes validated by either Sanger sequences or Illumina data (18S coding region only). Black lines represent the length of the haplotypes constructed by our program. Some haplotypes are spanning two or more subregions.

Using the number of Illumina reads mapped on the different haplotypes, it was possible to estimate the relative proportion of each haplotype. For each rDNA region, the number of reads per haplotype was estimated (Figure 5). In the 5′-IGS/ETS, 12 haplotypes were compared, including three haplotypes presenting a high proportion (more than 17.8% of the rDNA copies). Three haplotypes presented a very small number of estimated copies (between 0.01% and 2.50%). The estimated proportions for the six other haplotypes detected in this region were 6.92% on average. In the 18S coding region, two haplotypes were present in very different frequencies. The first one represented only 1.28% of the rDNA copies and the second one was overrepresented (98.72% of rDNA copies). In the ITS-1, two haplotypes were highly frequent (46.45% and 51.49% respectively). The third haplotype detected in the ITS-1 was represented in a low copy number (2.06%). In the 25S coding region, two haplotypes were approximately equally frequent (39.84% and 40.66%), and two others were less represented (4.86% and 14.65%). Finally, in the different regions studied, we found between one and three copies highly represented, which could indicate that these copies present a high number of repetitions in the *S. maritima* genome.

### rDNA location on *S. maritima* chromosomes

**Figure 6:**
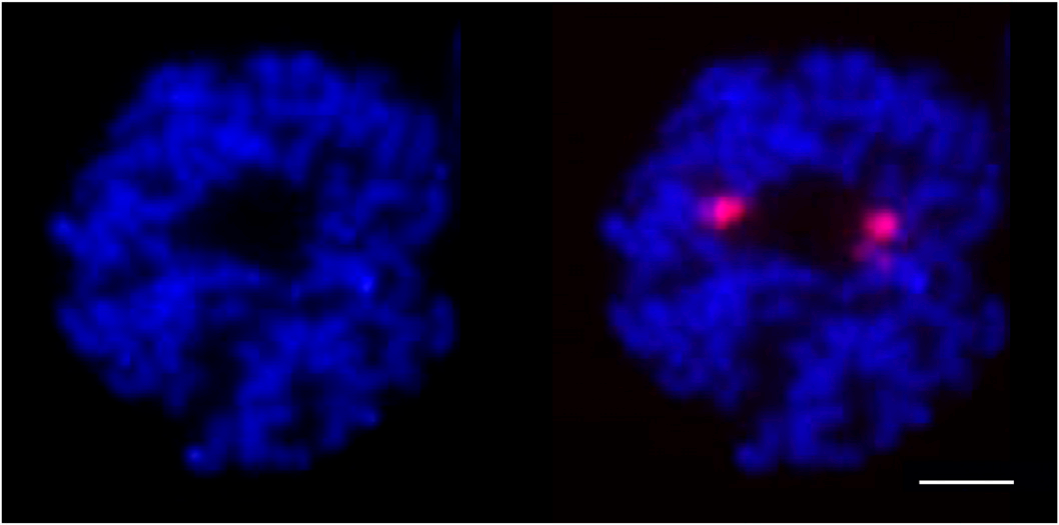
Fluorescence *in situ* hybridization (FISH) of the 45S rDNA in *S. maritima* on somatic metaphase chromosomes counterstained with DAPI (blue), in the absence (left) or presence of the red rDNA probe (right). Bar, 5 μm.

Fluorescence *in situ* hybridization (FISH) was performed from *S. maritima* somatic metaphase chromosomes (counterstained with DAPI) to identify the physical position of 45S rDNA arrays. The chromosomal locations of 45S rDNA arrays in *S. maritima* are shown in Figure 6. Hybridization signals were consistently observed on two chromosomes indicating the presence of one pair of 45S rDNA loci.

## Discussion

In this study, we developed a program for SNP and haplotype detection. This program was applied on Roche-454 pyrosequencing data corresponding to the 45S rDNA genes of *S. maritima*. The objective was to detect the different rDNA sequences (designated as haplotypes) in this hexaploid species where both paralogous and homeologous copies are expected for this gene family. Results were validated by cloning and (re)-sequencing and also from Illumina sequencing datasets; the results allowed the estimation of the relative proportion of haplotype variation. To date, very few programs or pipelines have been developed to distinguish duplicated copies from next-generation sequencing data without diploid parents as a reference. SNiPloid (Peralta *et al.* 2013), BamBam (Page *et al.* 2014), or HyLiTE (Duchemin *et al.* 2014) were developed for haplotype detection in polyploid species using comparisons between the polyploids and their diploid progenitors. The POLiMAPS program developed by Tennessen *et al.* (2014) used a related sequenced diploid genome combined with dense genetic map to infer homeologs in octoploid *Fragaria*. Our program will be particularly useful in nonmodel systems having experienced repeated duplication events and for which diploid reference genomes are not available.

### Number of rDNA loci in *S. maritima*

Molecular cytogenetic analysis by FISH revealed the presence of one pair of 45S rDNA signals on the chromosomes of *S. maritima* instead of the three expected pairs for a hexaploid genome. The number of rDNA loci varies greatly among flowering plants of the same ploidy level. For instance, only one locus was identified in diploid *Medicago* species (Rosato *et al.* 2008), whereas five loci were detected in diploid *Brassica rapa* species (Maluszynska and Heslop-Harrison 1993). In polyploids, the number of rDNA loci can be either retained (in recent or old polyploids) or deeply decreased, most probably as part of the diploidization process (Leitch and Bennett 2004). For example, three rDNA loci were identified in the hexaploid bread wheat (AABBDD), corresponding to the sum of the loci present in the parental genomes [two in the allotetraploid *Triticum turgidum* (AABB) and one in the diploid *Aegilops tauschii* (DD)] (Badaeva *et al.* 1996; El-Twab 2007). Similarly, the number of loci detected in the recent *Tragopogon* allotetraploids is additive with respect to their diploid parental species (Kovarik 2005). In contrast, in other polyploid genera, such as *Fragaria*, a decrease in the number of rDNA loci during the diploidization process is observed, with only six loci in the decaploid *F. iturupensis* (Liu and Davis 2011), whereas related diploid species exhibit three loci. In the hexaploid *S. maritima*, the presence of one locus indicates rDNA loci have been lost since its formation, following evolution of the tetraploid and hexaploid *Spartina* lineages that occurred in the past 6–10 mya (Rousseau-Gueutin *et al.* 2015). To our knowledge, this is the first 45S locus number reported in *Spartina*, and it would be interesting to examine this number in other polyploid *Spartina* species. Such rDNA locus loss can occur rapidly, as in the recent allotetraploid *Brassica napus*, which formed approximately 8000 years ago (Chalhoub *et al.* 2014), where six loci are retained instead of the seven expected from the progenitor species (five in *B. rapa* and two in *B. oleracea*) (Snowdon *et al.* 1997).

### *In silico* detection of rDNA domains

The intraindividual homogeneity of ribosomal genes was so far mainly examined across plant species by cloning and sequencing, a relatively time-consuming procedure that limits the number of sequences that can be sampled in the genome. Massively parallel sequencing now provides the opportunity to assess more accurately the intraindividual sequence polymorphism (Matyášek *et al.* 2012). Furthermore, in rDNA genes, most studies have focused on the ITS region (Alvarez and Wendel 2003; Poczai and Hyvönen 2010) or the 18S and 25S coding regions. For the first time, all the repeat unit and spacers (IGS and ETS) in *Spartina* are reported here. Analysis of the contig corresponding to the 45S ribosomal DNA unit of *S. maritima* enabled us to annotate the different regions of the transcriptional unit and to detect the different copies for each region (IGS/ETS; 18S; ITS-1; 5.8S; ITS-2; and 25S) using an approach relying on a low-stringency mapping of NGS reads.

Detection of the different domains was performed by sequence alignments of 45S rDNA available in the databases of two Poaceae species (maize and rice). Lengths of the rDNA coding regions and ITS of *S. maritima* are similar to those found in two Poaceae. However, the percentage of GC of the two ITS and 5.8S coding regions is very low in comparison with other Poaceae (Table 1). When examining the GC content of the ITS region in various grass species, we found that some Chloridoideae lineages related to *Spartina* in the Zoysieae, Cynodonteae, and Eragrostideae tribes similarly exhibit less than 60% GC content (Table S2). In more distantly related Chloridoideae tribes such as Triraphideae and Centropodieae (Soreng *et al.* 2015), as well as other grass subfamilies, the GC content is greater (up to 79.1% in rice ITS-2) (Table S2). Other monocot lineages (*e.g.*, *Prospero autumnale*, Hyacinthaceae) also exhibit higher GC content. Although information about more monocot representatives is needed, these results examined in the light of recent grass phylogenies (Soreng *et al.* 2015) suggest that GC content decrease occurred in the Chloridoideae lineage following the split between the basal Centropodieae–Triraphideae tribes and the other more derived tribes including *Spartina*.

Within 25S, variable regions were identified when comparing *S. maritima* with *Zea mays* and *Oryza sativa* (Figure 1). These regions mostly correspond to previously described expansion segments (Hancock and Dover 1988) representing variable 26S subdomains in plants (Kuzoff *et al.* 1998). The 5′-ETS detected in *Oryza sativa* (966 bp) has a similar length compared to the 5′-ETS of *Zea mays* (825 bp) identified by Toloczyki and Feix (1986). However, this spacer is much larger in *S. maritima* (1754 bp), with lower GC content (50.9%) than in other Poaceae (70.0% and 73.0% for maize and rice, respectively). These results are consistent with the literature showing that the size of 5′-ETS is variable across species, even within genera. Volkov *et al.* (1996) found different lengths of the 5′-ETS between two tobacco species: *Nicotiana sylvestris* and *Nicotiana tomentosiformis*, which have, respectively, an ETS size of 1444 bp and 2172 bp. The size difference between these two ETS is explained by the number of subrepeats present in region 2 of the ETS, where five and 10 subrepeats are present in *N. sylvestris* and *N. tomentosiformis*, respectively (Volkov *et al.* 1996). In the *Fabaceae* family, *Lupinus luteus* has a small 5′-ETS with a size of 487 bp (Mahé *et al.* 2010). It would be interesting to identify and compare the 5′-ETS in other hexaploid and tetraploid *Spartina* species as well as in related Chloridoids to explore the amplitude and origin of this size variation. These studies would verify whether this heterogeneity is widespread in the polyploid *Spartina* genus, or whether it is a characteristic of the hexaploid *Spartina maritima*.

### Polymorphism of coding and noncoding regions within the *S. maritima* rDNA locus

Thirty-nine polymorphic variants and 20 haplotypes were detected in *S. maritima* rDNA using our developed program. Thirty-four SNPs and 11 haplotypes were validated by cloning and Sanger sequencing or with Illumina data. The 5.8S coding region exhibited less heterogeneity than 18S and 25S coding regions. Indeed, no polymorphism was encountered in the 5.8S and only one polymorphic site (one deletion) was encountered in the 18S coding region, highlighting their high sequence homogeneity. The 25S unit also presents little variation, with two SNPs (variants presenting substitution and indel at the same position) detected using pyrosequencing data and validated by the other methods. Although most coding regions are highly conserved, some variation may be observed in the 18S region. Such results are in accordance with those of Mentewab *et al.* (2011), who detected a deletion of 270 bp within *Arabidopsis thaliana* 18S, or those obtained in animals (*e.g.*, *Anguilla*, for which a specific SNP was found) (Frankowski and Bastrop 2010). Similarly, the intraspecific variation of the 18S and 25S detected in *S. maritima* accords with the results obtained in two *Oenothera* species using cloning and sequencing methods (Seo *et al.* 2013), for which 17 and 10 SNPs were identified in the 18S of *O. odorata* and *O. laciniata* and 11 and 13 SNPs in the 25S regions, respectively.

In the noncoding regions, the internal transcribed spacers of *S. maritima* exhibit less heterogeneity than the IGS/ETS regions, without any polymorphism in ITS-2 and only one substitution/deletion in ITS-1. The high polymorphism encountered in the IGS/ETS region (25 variants in the 5′-IGS/ETS) is consistent with the literature, which indicates that intergenic regions evolve more rapidly than the ITS region, which is most particularly prone to homogenization (Alvarez and Wendel 2003; Kovarik *et al.* 2008). The high IGS/ETS variation we detected at the intraindividual level is in accordance with other studies at the interindividual or intraspecific levels (Poczai and Hyvönen 2010). Indeed, ETS regions evolve very quickly, 1.5-times faster than ITS regions (Bena *et al.* 1998); IGS regions also evolve rapidly (Chang *et al.* 2010). The number of detected haplotypes in the IGS/ETS region (13) exceeds the number of expected homeologous copies in a hexaploid species, which suggests incomplete homogeneization within the rDNA locus as also evidenced in diploid *Nicotiana* species (Matyášek *et al.* 2012). Our finding of a single rDNA locus in *Spartina maritima* mentioned above supports this hypothesis.

Like coding regions, the ITS region in *S. maritima* contains two haplotypes that are highly represented. The ITS region might be as strongly selected as the flanking coding regions, but the rapid evolution of the ITS region at the interspecific level suggests that this region is rather subject to more rapid homogenization (within individual genomes) than the other regions. Interspecies variation is then most likely more important than intraindividual (intragenomic variation), which explains why this region is useful in phylogenetic studies aiming at reconstructing organismal (*e.g.*, species) history (Baumel *et al.* 2002 for *Spartina*). In the 5′-IGS/ETS, three haplotypes are present in high proportion and two haplotypes are less represented, which could indicate that the homogenization process is underway in these regions.

### Impact of the parameters on polymorphism and haplotype detection

The number of SNPs and haplotypes that may be detected in our study is indeed limited by the employed technology (*i.e.*, 454 read depth) and the parameters used to avoid false positives resulting from sequencing errors. Thus, the number of actual rDNA variants and their relative proportion (or copy number) in the *S. maritima* genome is most likely underestimated. In our developed program, several parameters can be tuned for relaxing or constraining read mapping, SNP detection, and haplotype construction. For example, the choice to not consider nucleotides present in less with a frequency lower than 20% at a specific position is in line with the procedure usually used when analyzing polyploid genomes. In their analysis of allotetraploid cotton EST (Expressed Sequence Tag) assemblies, Udall *et al.* (2006) have chosen to set this parameter to 25%. Tennessen *et al.* (2014) have chosen to adjust this parameter to 12.5% (one-eighth frequency) to detect variants in octoploid *Fragaria* species. Our parameter is less stringent than the parameter used for homeo-SNP detection (fixed to 40%) in allotetraploid cotton (Page *et al.* 2013a,b). This lower value allowed us to detect putative homeologous and paralogous copies, as expected for highly repeated rDNA arrays. Using this parameter, 34 variants were detected and 29 were validated. Four of these variants are localized in homopolymeric regions of three or four repeats. The developed program does not consider the homopolymeric regions of at least five repeats. These results could indicate that variants in homopolymeric regions of three or four repeats correspond to false positives and should also not be considered. In the internal transcribed spacer and the coding region, it was possible to validate most of the haplotypes constructed by the developed program (seven haplotypes validated on nine). In the noncoding region, the number of haplotypes, their relative estimated proportion, and the cloning depth explain the number of haplotypes not validated by the cloning method. In fact, several haplotypes localized in the 5′-IGS/ETS built with the developed program were also detected and validated using cloning/(re)-sequencing in the *Spartina* hybrids between *S. maritima* and *S. alterniflora* (*S. x townsendii* and *S. x neyrautii*; unpublished data). New and emerging single molecule sequencing methods that are producing longer reads should provide precious information regarding the assembly of longer (or even whole-length) 45S rDNA haplotypes.

In summary, our program enables detection of SNPs and haplotypes within NGS read datasets. Haplotype construction and validation are based on three methods/technologies [Roche-454, cloning, and (re)-sequencing Sanger method and Illumina approaches] in the context of polyploidy in a nonmodel species where diploid reference parental genomes are not available. The program developed in this study could then be used for any polyploid species (including high ploidy levels) with or without reference genome sequences but also on diploid species (*e.g.*, for allele detection from amplicon data sets). In the polyploid *Spartina* genus, which contains various ploidy levels (4×, 6×, 7×, 9×, and 12×), such a program will be useful to detect duplicated homeologous genes and to increase our understanding of their origin and evolutionary fate.

## 
